# Altered Glucagon Response to Oral Glucose in Individuals at Different Stages of Type 1 Diabetes Development

**DOI:** 10.1210/clinem/dgaf601

**Published:** 2025-10-31

**Authors:** Helena Kontola, Luís Crisóstomo, Eliisa Löyttyniemi, Jaakko J Koskenniemi, Riitta Veijola, Jorma Toppari, Jukka Kero

**Affiliations:** Department of Pediatrics, Turku University Hospital, Turku FIN-20500, Finland; Medicity Research Laboratory, University of Turku, Turku FIN-20500, Finland; InFlames Research Flagship, Institute of Biomedicine, University of Turku, Turku FIN-20500, Finland; Department of Biostatistics, University of Turku and Turku University Hospital, Turku FIN-20500, Finland; Department of Pediatrics, Turku University Hospital, Turku FIN-20500, Finland; Health Informatics Institute, University of South Florida, Tampa, FL 33512, USA; Department of Children and Adolescents, Oulu University Hospital, Oulu FIN-90220, Finland; Department of Pediatrics, Research Unit of Clinical Medicine, Medical Research Center, University of Oulu, Oulu FIN-90220, Finland; Department of Pediatrics, Turku University Hospital, Turku FIN-20500, Finland; Research Centre for Integrative Physiology and Pharmacology, and Centre for Population Health Research, InFlames Research Flagship, Institute of Biomedicine, University of Turku, Turku FIN-20500, Finland; Department of Pediatrics, Turku University Hospital, Turku FIN-20500, Finland; Department of Clinical Sciences, Faculty of Medicine, University of Turku, Turku FIN-20500, Finland

**Keywords:** pancreatic islet, α cells, prediabetes, hyperglucagonemia

## Abstract

**Context:**

Autoimmune destruction of β cells and their functional decline precedes the clinical onset of type 1 diabetes. However, altered α-cell function and hyperglucagonemia may contribute to the development of hyperglycemia and ketoacidosis at onset.

**Objective:**

In this cross-sectional study, we analyzed glucagon concentrations during an oral glucose tolerance test (OGTT) in individuals at the early stages of type 1 diabetes to understand the role of α-cell function in the disease process.

**Methods:**

We recruited 47 participants, aged 4 to 25 years, from the Finnish Diabetes Prediction and Prevention (DIPP) study, and categorized them into the following groups: islet autoantibody (IAb) negative, single IAb positive, and stages 1 to 3 of type 1 diabetes. Glucagon levels were measured during a 6-point OGTT using a conventional radioimmunoassay, alongside insulin, C-peptide, glucose, and glucagon-like peptide-1 (GLP-1).

**Results:**

Fasting plasma glucagon levels increased with disease progression. The longitudinal patterns of glucagon concentrations during the OGTT differed significantly between groups, with a paradoxical 15-minute glucagon increase observed only in individuals at early stage 3 of type 1 diabetes.

**Conclusion:**

These findings highlight the need for prospective studies to further elucidate the role of α cells in disease progression and support testing pharmacotherapies aimed at improving both α- and β-cell functions during disease development.

The progressive decline of β-cell function in the pancreatic islets leads to type 1 diabetes, resulting in lifelong dependency on exogenous insulin and glucose monitoring. This immune-mediated β-cell impairment originates from islet inflammation and leads to the early development of autoantibodies (1, 2). Based on islet autoantibodies (IAbs) and glucose metabolism, type 1 diabetes progresses through distinct presymptomatic stages (3): stage 1, with 2 or more IAbs and normoglycemia; stage 2, with multiple IAbs and dysglycemia; and stage 3, meeting the diagnostic criteria for diabetes, which can be further subdivided into stage 3a and 3b, based on residual β-cell function or disease duration (4).

Beyond β-cell loss, α-cell dysfunction in diabetes was recognized more than 50 years ago (5), with subsequent studies reporting glucagon excess in type 1 diabetes (6). In rodent models, deleting the glucagon receptor or inhibiting hyperglucagonemia prevents diabetes (7). Mechanistically, hyperglucagonemia in type 1 diabetes aligns with the intraislet hypothesis, which suggests that intraislet insulin inhibits glucagon release (8). β-Cell loss and reduced insulin production may elevate glucagon, stimulating hepatic gluconeogenesis and glycogenolysis, increasing plasma glucose levels. Glucagon secretion is regulated by glucose, insulin, somatostatin from the δ cells and incretins, including glucagon-like peptide-1 (GLP-1), glucagon-like peptide-2, and glucose-dependent insulinotropic polypeptide, responding to plasma glucose, food intake, and exercise (6, 9, 10).

Compared to β-cell function, α-cell dysfunction in early stages of type 1 diabetes is poorly understood. In healthy individuals, rising plasma glucose suppresses glucagon secretion via insulin and other intraislet signals. In type 1 diabetes, glucagon regulation fails, potentially causing abnormal glucagon responses to hypoglycemia, glucose boluses, or meals (11). Progressive hyperglucagonemia (12, 13) has been observed after diagnosis, and abnormal α-cell function has been reported in individuals with dysglycemia as well as in IAb-positive adults (14, 15). Previous studies have suggested that glucagon could be more involved in the disease process than previously acknowledged (16). Moreover, the genetic expression of α cells is altered in type 1 diabetes, while the gene expression profile remains normal in the remaining β cells (17). However, whether glucagon secretion is affected in the early stages of diabetes remains unclear.

We hypothesized that the glucagon response to oral glucose is altered in the early stages of type 1 diabetes. Therefore, we aimed to elucidate glucagon concentration patterns in children and adolescents during the early stages of the disease.

## Materials and Methods

### Study Participants

Participants were recruited between June 2020 and November 2021 from the Finnish Type 1 Diabetes Prediction and Prevention (DIPP) study, a prospective birth cohort study (ClinicaTrials.gov No. NCT3269084). Children with human leukocyte antigen–conferred risk for type 1 diabetes were followed regularly (18, 19), with islet autoantibody (IAb) screening against insulin (IAA), glutamate decarboxylase (GADA), insulinoma-associated antigen 2 (IA-2A), and zinc transporter 8 at 3, 6, 12, 18, and 24 months, and annually until age 15 years (19-22). After seroconversion, visits occurred every 3 months (23). Individuals with 2 IAbs or greater underwent oral glucose tolerance tests (OGTTs) and glycated hemoglobin A_1c_ (HbA1c) measurements every 3 to 6 months.

Participants were categorized into 5 groups based on OGTT and IAb results (Supplementary Fig. S1 (31)) as previously described (24, 25): (1) no IAbs and normal glucose metabolism (0 IAb); (2) 1 IAb and normal glucose metabolism (1 IAb); (3) multiple IAbs and normal glucose metabolism (stage 1); (4) multiple IAbs and dysglycemia (stage 2); and (5) early stage 3 type 1 diabetes based on 2 consecutive OGTTs.

This substudy used the same cohort as Kontola et al (24) on continuous glucose monitoring. Power calculations were based on mean evening glucose as the primary outcome. We excluded pregnant individuals and individuals with body mass index (BMI), or ISO-BMI, greater than 35. In addition, prior to the OGTT, all individuals had to be asymptomatic, healthy, and not receiving any treatment that would affect glucose metabolism.

This study was approved by the ethics committee of the Hospital District of Northern Ostrobothnia, Oulu, Finland (T08/034/19). Written informed consent was obtained from participants and/or their guardians.

### Laboratory Analysis

#### Oral glucose tolerance test

Six-point OGTTs (23) were conducted between 8 and 10 Am with blood samples collected at 0, 15, 30, 60, 90, and 120 minutes. Participants fasted overnight (>10 hours) before ingesting 1.75 g/kg glucose (max 75 g for those > 43 kg, Glucosepro, Mediq) within 5 minutes.

#### Biochemical measurements

Plasma insulin, C-peptide, and HbA_1c_ were measured using electrochemiluminescence immunoassays (ECLIA, Roche Diagnostics) at Turku University Hospital: Glucose was analyzed enzymatically. HbA_1c_ values were converted to percentages using the American Diabetes Association converter (https://ngsp.org/convert1.asp).

Blood was collected into precooled EDTA tubes, kept on ice, centrifuged (10 minutes, 2000*g*, 4 °C) within 15 minutes, plasma aliquoted, and stored at −20 °C or −80 °C for long-term storage.

#### Glucagon measurement

Glucagon was quantified using a validated radioimmunoassay (EURIA-Glucagon, Euro Diagnostica AB) at an accredited laboratory (Vita Laboratories). The assay correlates with World Health Organization standard 69/194 and uses an antiserum (26, 27) that specifically recognizes pancreatic glucagon (1-29) with low cross-reactivity (<0.1% with GLI/GLP-1; <0.7% with oxyntomodulin). Intra-assay coefficient of variation ranged from 4.8% to 8.1%, total variation 3.9% to 8.3%, and recovery 97.6%. The assay has been validated against other methods and shows expected fasting glucagon levels (<60 pmol/L/206 ng/L) in healthy individuals and decreased responses during OGTT (28, 29).

#### Islet autoantibodies

IAA, IA-2A, and GADA were analyzed at the DIPP and PEDIA laboratories as previously described (30). Glucagon, insulin, C-peptide, and active GLP-1 were also measured using a MILLIPLEX kit (HDIAB-34K-PMX5, RRID:AB 3717320, Sigma Aldrich) for comparison.

### Statistical Analysis

Changes in mean glucagon, C-peptide, and insulin values over OGTT time points were compared using a linear mixed-effects model for repeated measurements, with time (within, categorical), stage (between), and their interactions as fixed effects. Participant was included as a random effect with a compound symmetry covariance structure. Analyses were performed using IBM SPSS Statistics version 29 (IBM Corp) and SAS v9.4 (SAS Institute Inc). Figures were generated using GraphPad Prism v10.1.0 (GraphPad Software).

Normality was assessed using D’Agostino Pearson and Shapiro-Wilk tests with QQ-plots, variance equality with the Brown-Forsythe test; a one-way analysis of variance with Tukey post hoc test was used for group comparisons; and the Fisher exact test for analyzing frequencies. Skewed C-peptide and insulin values were log-transformed. Baseline glucose medians (Table 1) remained nonnormal after transformation and were analyzed using the Kruskal-Wallis test.

**Table 1.** Data are shown as mean and SD unless stated otherwise

Demographics of study participants							
Group	Total	0 IAb	1 IAb	Stage 1	Stage 2	Stage 3	*P*
Sex (male), N	46 (16)	8 (1)	6 (2)	12 (6)	12 (3)	8 (4)	.38
Age (range), y	11.7	9.3	10.8	11.3	14.1	11.6	.30
	(3.9-25.4)	(3.9-15.2)	(7.4-13.1)	(5.1-25.4)	(4.0-20.2)	(4.5-19.7)	
HbA_1c_, mmol/mol	36.76 (5.45)	*35.83 (3.19)* ^a^	*35.00 (2.83)^a^*	*32.45 (2.02)^a^*	*36.40 (3.24)^a^*	45.13 (5.00)	<.0001
HbA_1c_, %	5.5 (0.5)	*5.4 (0.3)^a^*	*5.4 (0.2)^a^*	*5.1 (0.2)^a^*	*5.5 (0.3)^a^*	6.3 (0.5)	<.0001
Time from seroconversion, range, y	7.5	No islet autoantibodies	5.6	7.9	7.8	8.0	.79
	(0.0-20.9)		(0.4-11.4)	(2.0-20.9)	(0.8-15.0)	(0.0-16.3)	
ISO-BMI**	22.40(2.66)	22.05(2.66)	22.30(2.95)	22.80(3.28)	22.46(2.31)	21.83(3.12)	.99

0 IAb and 1 IAb denote participants with 0 and 1 islet autoantibodies, respectively. Stage 1 individuals have 2 or more islet autoantibodies and normal glucose tolerance, stage 2 have 2 or more islet autoantibodies and dysglycemia, and stage 3 participants have early asymptomatic type 1 diabetes. *P* values indicate the analysis of variance results from testing group means.

Abbreviations: BMI, body mass index; HbA_1c_, glycated hemoglobin A_1c_.

^*a*^Indicates a statistically significant difference (*P* < .005) between the denoted group and stage 3.

**ISO-BMI is used to evaluate children's and adolescents' BMI instead of regular BMI. ISO-BMI takes into account sex and age. Regular BMI was used for participants older than 18 years; their BMIs were between 21.7 and 26.7, and they were not included in this table.

## Results

### Study Participants

An overview of the study and participant classification is shown in Supplementary Fig. S1 (31). The 47 participants (aged 4-25 years) were healthy and asymptomatic, with BMI or ISO-BMI within the normal range and no differences in pubertal status between the groups. Five individuals were older than 18 years, and their BMIs were between 21.7 and 26.7. They were not included in the ISO-BMI data in Table 1. Mean HbA_1c_ ranged from 32 to 45 mmol/mol (5.1% 6.3%), increasing progressively from stage 1 to 3. It was lower in the 0 IAb and 1 IAb groups compared to stage 1. In stage 3 (pre insulin treatment), the mean HbA_1c_ was 45.1 mmol/mol (range, 39-53 mmol/mol; 5.7%-7.0%), consistent with very early stage 3 type 1 diabetes (see Table 1). Information about comorbidities in addition to type 1 diabetes was collected. One individual in the stage 2 group had autoimmune hypothyroidism. Two individuals had celiac disease, one in stage 2 and one in stage 3. Four individuals had asthma, one in each group except the 1 IAb group. All of these individuals were asymptomatic and had well-controlled treatment, and there were no statistically significant differences in disease frequencies between study groups.

### Glucagon Concentration During Oral Glucose Tolerance Test

**Figure 1.**
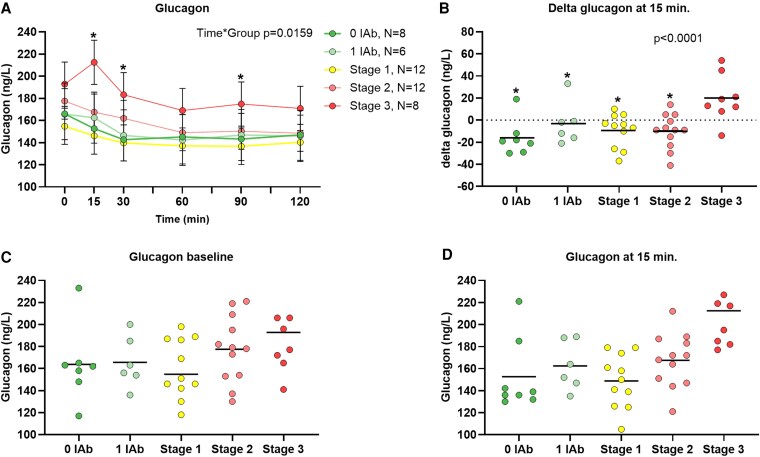
Glucagon response during an oral glucose tolerance test (OGTT) in participants at various early stages of type 1 diabetes. Panel A displays the means with a 95% CI for glucagon (ng/mL) during the OGTT at distinct time points. Participants are grouped by autoantibody status and disease stage: those with one islet autoantibody (1 IAb), those without any autoantibodies (0 IAb), and stages 1-3 of type 1 diabetes (as indicated in the legend). “N” denotes the number of participants. Panel B shows covariance analysis results of the glucagon difference between 0 and 15-minute time points. The mean difference is shown by horizontal lines and individual values are indicated by data points. Panels C and D show individual raw glucagon values during OGTT at baseline before oral glucose bolus and at 15 minutes after the glucose bolus, respectively. The stage 3 group consists of asymptomatic participants at the time of diabetes diagnosis, before the start of insulin treatment. The *P* value indicates the statistically significant difference between groups over time as analyzed by the linear mixed-effects model. The asterisk (*) in panel A indicates a statistically significant difference between stage 3 and the 0 IAb group during the OGTT in linear mixed-effects model analysis. The asterisk (*) in panel B indicates a statistically significant difference between stage 3 and the denoted group.

Modeled glucagon responses during OGTT across type 1 diabetes stages are shown in Fig. 1. The glucagon trajectory over 2 hours differed significantly between groups (*P* = .0159 for group-by-time interaction). Fasting glucagon was highest in stage 3, with a statistically significant difference between stages 3 and 1 (mean difference 38 ng/L [95% CI, 12.2-63.8 ng/L]; *P* < .01) (Table 2, Supplementary Table S1 (31)). In the linear mixed-effects model, glucagon concentrations were significantly higher at all time points in stage 3 compared to stage 1. The greatest glucagon increase occurred in the stage 3 group, with 7 out of 8 individuals showing an inappropriate rise at 15 minutes post glucose bolus. At this time point, stage 3 differed significantly from all the other groups (Fig. 1A and 1B, Supplementary Table S1 (31)). We also conducted a correlation analysis of glucagon and glucose level changes between consecutive time points, but no statistically significant correlations were found.

**Table 2.** Glucagon means with 95% CI during an oral glucose tolerance test from linear mixed-effects model analysis

Glucagon means with 95% CI during OGTT, ng/L					
Groups	0 IAb	1 IAb	Stage 1	Stage 2	Stage 3
Time, min					
0	166.0	165.7	154.8	177.5	192.8
	(145.7-186.3)	(142.6-188.7)	*(138.5-171.1)^a^*	*(161.2-193.8)*	(172.8-212.7)
15	152.6	162.5	*146.1*	*167.5*	212.5
	*(132.6-172.6)^a^*	*(139.5-185.6)^a^*	*(129.6-162.6)^a^*	*(151.2-183.8)^a^*	(192.5-232.5)
30	*142.6*	*146.5*	*139.7*	161.9	183.3
	*(122.7-162.6)^a^*	*(123.5-169.6)^a^*	*(123.4-156.0)^a^*	(145.6-178.2)	(163.3-203.2)
60	*145.1*	*142.3*	*137.0*	149.0	169.0
	(125.2-165.1)	(119.3-165.4)	*(120.7-153.3)^a^*	(132.7-165.3)	(149.0-189.0)
90	143.3	146.8	*136.7*	150.2	174.9
	(123.3-163.2)*^a^*	(123.8-169.9)	*(120.3-153.2)^a^*	(134.0-166.5)	(154.9-194.8)
120	147.0	146.0	*140.3*	148.5	170.9
	(127.0-167.0)	((123.0-169.1)	*(124.0-156.6)^a^*	(132.2-164.8)	(150.9-190.8)

Time × Group *P* = .0159.

0 IAb and 1 IAb refer to individuals with 0 and 1 islet autoantibodies, respectively. Stages 1, 2, and 3 represent different stages of type 1 diabetes. Stage 1 has 2 or more islet autoantibodies and normal glucose tolerance, stage 2 has 2 or more islet autoantibodies and dysglycemia, and stage 3 individuals have early asymptomatic type 1 diabetes.

^*a*^Indicates a statistically significant difference between stage 3 and denoted group. All pairwise comparison *P* values are presented in Supplementary Table S1 (31).

### Glucose, Insulin and C-Peptide Responses During Oral Glucose Tolerance Test

**Figure 2.**
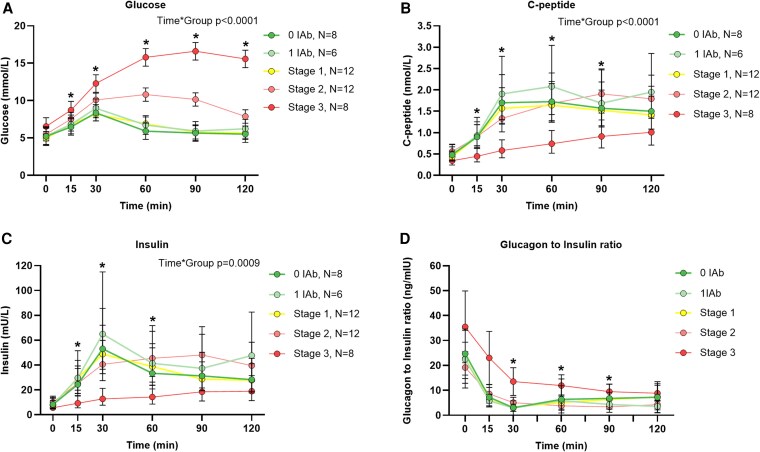
Glucose, insulin, and C-peptide levels and glucagon insulin ratio during an oral glucose tolerance test (OGTT) in participants at various early stages of type 1 diabetes. Means of A, glucose (mmol/L); B, C-peptide; and C, insulin levels; and D, glucagon-to-insulin raw value ratio during the OGTT. Means were analyzed using the linear mixed-effects model. *P* values indicate statistically significant differences between groups over time. Error bars show 95% CI, with colored circles representing means. Light green circles represent participants with one islet autoantibody (1 IAb), while those without any (0 IAb) are shown with dark green circles. Stages 1 to 3 of type 1 diabetes are depicted as follows: yellow circle for stage 1, light red for stage 2, and red for stage 3 individuals. The asterisk (*) in panels A, B, and C indicates a statistically significant difference between the stage 3 and 0 IAb groups during the OGTT. In panel D, the asterisk (*) indicates a statistically significant difference between group means. “N” denotes the number of participants. More in-depth analysis results are shown in Table 2, with additional parameters presented in Supplementary Tables S1 to S5 (31).

Fig. 2 presents glucose, insulin, and C-peptide responses during the OGTT. The most notable differences were between stage 3 and all other groups. Pairwise comparison at all time points are presented in Supplementary Table S1 (31).

Glucose peaked at 60 minutes in stage 2 and 90 minutes in stage 3, whereas in other groups it peaked at 30 minutes (Supplementary Table S2 (31)). Insulin and C-peptide were lower in stage 2 and 3 (see Fig. 2 and Supplementary Tables S1-S4 (31)). Glucagon-to-insulin ratio was highest in stage 3 at all time points, with statistically significant differences compared to the 0 IAb and 1 IAb groups at 30 minutes, and between stage 2 and 3 at 60 and 90 minutes (Supplementary Table S5 (31), Fig. 2).

### Glucagon-like Peptide-1 Concentrations During Oral Glucose Tolerance Test at Different Stages of Type 1 Diabetes

To explore whether GLP-1 contributed to glucagon dysregulation, we assessed active GLP-1 concentrations during an OGTT. Levels were highest in the 0 IAb and 1 IAb groups, but no statistically significant differences were observed between groups (*P* = .36; Supplementary Fig. S2, Table S6 (31)). A positive correlation between GLP-1 and glucagon was found at 30 (*r* = 0.70; *P* = .01) and 60 minutes (*r* = 0.72; *P* = .020) in stage 1, and at 60 (*r* = 0.80; *P* = .01) and 120 minutes (*r* = 0.65; *P* = .04) in stage 2.

## Discussion

While β-cell decline is central to type 1 diabetes, this study highlights that increased plasma glucagon levels may contribute to early disease stages, offering insights into pathogenesis and potential therapeutic targets. Using the OGTT to simulate real-world postprandial metabolism, we identified early abnormalities in glucagon regulation. To our knowledge, plasma glucagon responses during OGTT have not been examined at such early prediabetes stages.

A paradoxical rise in glucagon occurred in asymptomatic individuals at early stage 3 type 1 diabetes, before insulin therapy. Mild fasting hyperglucagonemia was also observed prior to stage 3, suggesting inappropriate glucagon secretion begins before clinical disease onset. Simultaneous decreases in insulin secretion in stage 2 and loss of the early insulin peak in stage 3 were evident. The reduced insulin response in stages 2 and 3 can lead to blunted or delayed glucagon suppression, resulting in hyperglucagonemia. According to the intraislet insulin hypothesis, reduced insulin indirectly promotes glucagon release via α-cell dysregulation. Hyperglucagonemia was defined in relation to the other stages and/or values exceeding normal range (>209 ng/L). In individuals with newly diagnosed type 1 diabetes, hyperglucagonemia has also been reported, with exaggerated mixed-meal responses worsening over time (13), supporting the bihormonal hypothesis that both insulin deficiency and hyperglucagonemia contribute to hyperglycemia progression (32).

Our results also support the notion that hyperglucagonemia is not simply driven by exogenous insulin. Kramer et al (33) reported a paradoxical increase in glucagon following a glucose bolus in individuals with longstanding type 1 diabetes that was not corrected by restoring euglycemia prior to testing. Furthermore, glucagon response remains abnormal even after the restoration of local insulin secretion (34).

Hyperglucagonemia and impaired postprandial glucagon suppression, well established in type 2 diabetes, may involve liver dysfunction affecting the liver–α-cell axis (35). In type 1 diabetes, reduced insulin and increased somatostatin (36) as well as intrinsic α-cell changes or disrupted paracrine regulation (37), may contribute to glucagon dysregulation, but these mechanisms were not evaluated in our study.

The cause of α-cell dysfunction remains unclear. Is it a consequence of β-cell autoimmunity, disrupted α-β-δ-cell interactions, hyperglycemia, reduced intraislet insulin, or impaired incretin signaling? In our study, paradoxical glucagon increases were seen only in early stage 3 individuals with the lowest insulin levels, emphasizing the role of intraislet insulin in glucagon regulation. Moreover, our study revealed that changes in glucagon levels during the OGTT do not appear in IAb-positive individuals unless they have already developed dysglycemia or type 1 diabetes. This would indicate that α-cell dysfunction is not caused by islet autoimmunity alone. Previous animal studies have demonstrated that glucagon stimulates insulin secretion through glucagon- and GLP-1 receptors located on the β-cell surfaces (38). While hyperglucagonemia in newly diagnosed patients has been attributed to inadequate insulin therapy (9), our findings show inappropriate glucagon responses and mild fasting hyperglucagonemia before insulin initiation. On the other hand, in individuals with established type 1 diabetes, postprandial elevations in glucagon appear independent of residual β-cell function, as shown by similar glucagon responses regardless of disease duration or fasting C-peptide levels (39). In healthy individuals, glucagon secretion is suppressed in response to an increase in insulin secretion leading to normal glucose homeostasis. In our study, glucagon levels increased from the 0- to 15-minute time point in individuals with asymptomatic type 1 diabetes. Could our findings be explained by the attempt to stimulate insulin via glucagon secretion (38, 40)? Could this also explain the positive correlation between GLP-1 and glucagon secretion in stages 1 and 2? Beyond insulin and hyperglycemia, incretins such as GLP-1 regulate α and β cells simultaneously (41). In our study, similar to prior studies, GLP-1 levels did not differ statistically significantly between groups (42, 43). A trend toward lower GLP-1 levels during the OGTT was observed in individuals at stages 1, 2, and 3, suggesting a possible decline in GLP-1 as type 1 diabetes progresses and individuals develop multiple IAbs. A preserved incretin response in the 0 and 1 IAb groups was detected. To clarify whether GLP-1 is altered early in the disease process during the OGTT, larger studies in early-stage individuals with normal glucose metabolism are warranted. However, a diminished incretin effect has been demonstrated in individuals with type 2 diabetes even in physiological levels of glucose-dependent insulinotropic polypeptide and GLP-1 (44).

This study has several limitations. The relatively small cohort may limit statistical power and warrants replication in larger studies. We would also like to acknowledge the exploratory nature of these glucagon findings, because glucagon was a secondary outcome in our power calculations. Multiple groups and time points increase the risk of type 1 error, though predefined contrasts were used. All participants had a high genetic risk of type 1 diabetes, potentially limiting generalizability. The cross-sectional design precludes conclusions on disease progression. Finally, glucagon was measured via conventional radioimmunoassay, which is less sensitive than newer enzyme-linked immunosorbent assays and may have reduced our ability to detect subtle group differences (28, 29).

In summary, we show that paradoxical glucagon responses occur before the clinical onset of diabetes and initiation of insulin treatment. Consistent with previous studies, fasting glucagon levels are modestly elevated in stage 2 compared to earlier stages (15). These findings underscore the need for prospective studies to clarify the role of α cells and GLP-1 secretion in early disease and support the exploration of therapies targeting both α- and β-cell function in the development of type 1 diabetes.

## Data Availability

The datasets generated during and/or analyzed during the current study are available from the corresponding author on reasonable request.

## Abbreviations

BMI: body mass index
DIPP: Finnish Diabetes Prediction and Prevention
GADA: glutamate decarboxylase
GLP-1: glucagon-like peptide-1
HbA_1c_: glycated hemoglobin A_1c_
IA-2A: insulinoma-associated antigen 2
IAA: screening against insulin
IAb: islet autoantibody
OGTT: oral glucose tolerance test

## References

[1] Bluestone JA, Herold K, Eisenbarth G. Genetics, pathogenesis and clinical interventions in type 1 diabetes. Nature. 2010;464(7293):1293‐1300. 20432533 10.1038/nature08933 PMC4959889

[2] Atkinson MA, Von Herrath M, Powers AC, Clare-Salzler M. Current concepts on the pathogenesis of type 1 diabetes-considerations for attempts to prevent and reverse the disease. Diabetes Care. 2015;38(6):979‐988. 25998290 10.2337/dc15-0144 PMC4439528

[3] Insel RA, Dunne JL, Atkinson MA, et al Staging presymptomatic type 1 diabetes: a scientific statement of jdrf, the Endocrine Society, and the American diabetes association. Diabetes Care. 2015;38(10):1964‐1974. 26404926 10.2337/dc15-1419 PMC5321245

[4] Tatovic D, Narendran P, Dayan CM. Perspective check for updates A perspective on treating type 1 diabetes mellitus before insulin is needed. Nat Rev Endocrinol. 2023;19(6):361‐370. 36914759 10.1038/s41574-023-00816-5

[5] Müller WA, Faloona GR, Aguilar-Parada E, Unger RH. Abnormal alpha-cell function in diabetes. Response to carbohydrate and protein ingestion. N Engl J Med. 1970;283(3):109‐115. 4912452 10.1056/NEJM197007162830301

[6] Sandoval DA, D’alessio DA. Physiology of proglucagon peptides: role of glucagon and GLP-1 in health and disease. Physiol Rev. 2015;95(2):513‐548. 25834231 10.1152/physrev.00013.2014

[7] Lee Y, Wang MY, Du XQ, Charron MJ, Unger RH. Glucagon receptor knockout prevents insulin-deficient type 1 diabetes in mice. Diabetes. 2011;60(2):391‐397. 21270251 10.2337/db10-0426 PMC3028337

[8] Kawamori D, Kurpad AJ, Hu J, et al Insulin signaling in α cells modulates glucagon secretion in vivo. Cell Metab. 2009;9(4):350‐361. 19356716 10.1016/j.cmet.2009.02.007 PMC2694613

[9] Wewer Albrechtsen NJ, Holst JJ, Cherrington AD, et al 100 years of glucagon and 100 more. Diabetologia. 2023;66(8):1378‐1394. 37367959 10.1007/s00125-023-05947-y PMC13288116

[10] Gerich JE, Lorenzi M, Bier DM, et al Prevention of human diabetic ketoacidosis by somatostatin. N Eng J Med. 2010;292(19):985‐989. 10.1056/NEJM197505082921901 804137

[11] Bisgaard Bengtsen M, Møller N. Mini-review: glucagon responses in type 1 diabetes—a matter of complexity. Physiol Rep. 2021;9(16):e15009. 34405569 10.14814/phy2.15009 PMC8371343

[12] Brown RJ, Slnaii N, Rother KI. Too much glucagon, too little insulin: time course of pancreatic islet dysfunction in new-onset type 1 diabetes. Diabetes Care. 2008;31(7):1403‐1404. 18594062 10.2337/dc08-0575 PMC2453684

[13] Sherr J, Tsalikian E, Fox L, et al Evolution of abnormal plasma glucagon responses to mixed-meal feedings in youth with type 1 diabetes during the first 2 years after diagnosis. Diabetes Care. 2014;37(6):1741‐1744. 24696460 10.2337/dc13-2612 PMC4030093

[14] Doliba NM, Rozo AV, Roman J, et al α cell dysfunction in islets from nondiabetic, glutamic acid decarboxylase autoantibody–positive individuals. J Clin Invest. 2022;2(11):e156243. 10.1172/JCI156243 PMC9151702 35642629

[15] Færch K, Vistisen D, Pacini G, et al Insulin resistance is accompanied by increased fasting glucagon and delayed glucagon suppression in individuals with normal and impaired glucose regulation. Diabetes. 2016;65(11):3473‐3481. 27504013 10.2337/db16-0240

[16] Unger RH, Cherrington AD. Glucagonocentric restructuring of diabetes: a pathophysiologic and therapeutic makeover. J Clin Invest. 2012;122(1):4‐12. 22214853 10.1172/JCI60016 PMC3248306

[17] Brissova M, Haliyur R, Saunders D, et al α cell function and gene expression are compromised in type 1 diabetes. Cell Rep. 2018;22(10):2667‐2676. 29514095 10.1016/j.celrep.2018.02.032 PMC6368357

[18] Ilonen J, Reijonen H, Herva E, et al Rapid HLA-DQB1 genotyping for four alleles in the assessment of risk for IDDM in the Finnish population. Diabetes Care. 1996;19(8):795‐800. 8842593 10.2337/diacare.19.8.795

[19] Kupila A, Muona P, Simell T, et al Feasibility of genetic and immunological prediction of type I diabetes in a population-based birth cohort. Diabetologia. 2001;44(3):290‐297. 11317658 10.1007/s001250051616

[20] Kukko M, Kimpimäki T, Korhonen S, et al Dynamics of diabetes-associated autoantibodies in young children with human leukocyte antigen-conferred risk of type 1 diabetes recruited from the general population. J Clin Endocrinol Metab. 2005;90(5):2712‐2717. 15872335 10.1210/jc.2004-1371

[21] Savola K, Bonifacio E, Sabbah E, et al IA-2 antibodies ± a sensitive marker of IDDM with clinical onset in childhood and adolescence. Diabetologia. 1998;41(4):424‐429. 9562346 10.1007/s001250050925

[22] Savola K, Sabbah E, Kulmala P, Vähäsalo P, Ilonen J, Knip M. Autoantibodies associated with type I diabetes mellitus persistafter diagnosis in children. Diabetologia. 1998;41(11):1293‐1297. 9833935 10.1007/s001250051067

[23] Hekkala AM, Ilonen J, Toppari J, Knip M, Veijola R. Ketoacidosis at diagnosis of type 1 diabetes: effect of prospective studies with newborn genetic screening and follow up of risk children. Pediatr Diabetes. 2018;19(2):314‐319. 28544185 10.1111/pedi.12541

[24] Kontola H, Alanko I, Koskenniemi JJ, et al Exploring minimally invasive approach to define stages of type 1 diabetes remotely. Diabetes Technol Ther. 2022;24(9):655‐665. 35653748 10.1089/dia.2021.0554

[25] American Diabetes Association Professional Practice Committee . 2. diagnosis and classification of diabetes: standards of care in diabetes-2025. Diabetes Care. 2025;48(Supplement_1):27‐49.

[26] Von Schenck H, Vasquez B, Unger RH. Lack of a “big plasma glucagon” response during arginine stimulation of glucagon secretion. Horm Metab Res. 1982;14(2):69‐71. 7068099 10.1055/s-2007-1018926

[27] Unger RH . Radioimmunoassay of glucagon. Metabolism. 1973;22(8):979‐985. 4581038 10.1016/0026-0495(73)90215-1

[28] Matsuo T, Miyagawa JI, Kusunoki Y, et al Postabsorptive hyperglucagonemia in patients with type 2 diabetes mellitus analyzed with a novel enzyme-linked immunosorbent assay. J Diabetes Investig. 2016;7(3):324‐331. 10.1111/jdi.12400 PMC4847885 27330717

[29] Rasmussen C, Richter MM, Jensen NJ, et al Preanalytical impact on the accuracy of measurements of glucagon, GLP-1 and GIP in clinical trials. Scand J Clin Lab Invest. 2023;83(8):591‐598. 38127365 10.1080/00365513.2023.2294470

[30] Salonen KM, Ryhänen S, Härkönen T, Ilonen J, Knip M. Autoantibodies against zinc transporter 8 are related to age, metabolic state and HLA DR genotype in children with newly diagnosed type 1 diabetes. Diabetes Metab Res Rev. 2013;29(8):646‐654. 23861236 10.1002/dmrr.2440

[31] Kontola H. Crisóstomo L, Löyttyniemi E, et al 2025. Data from: Altered Glucagon Response to Oral Glucose in Individuals at Different Stages of Type 1 Diabetes Development. Zenodo Digital Repository. 10.5281/zenodo.17350014. Deposited 14. October 2021. PMC13099181 41172278

[32] Unger RH, Eisentraut AM, Mccall MS, Keller S, Lanz HC, Madison LL. Glucagon antibodies and their use for immunoassay for glucagon. Proc Soc Exp Biol Med. 1959;102(3):621‐623. 13840405 10.3181/00379727-102-25338

[33] Kramer CK, Borgoño CA, Van Nostrand P, Retnakaran R, Zinman B. Glucagon response to oral glucose challenge in type 1 diabetes: lack of impact of euglycemia. Diabetes Care. 2014;37(4):1076‐1082. 24241790 10.2337/dc13-2339

[34] Paty BW, Ryan EA, Shapiro AMJ, Lakey JRT, Robertson RP. Intrahepatic islet transplantation in type 1 diabetic patients does not restore hypoglycemic hormonal counterregulation or symptom recognition after insulin independence. Diabetes. 2002;51(12):3428‐3434. 12453896 10.2337/diabetes.51.12.3428

[35] Capozzi ME, D’Alessio DA, Campbell JE. The past, present, and future physiology and pharmacology of glucagon. Cell Metab. 2022;34(11):1654‐1674. 36323234 10.1016/j.cmet.2022.10.001 PMC9641554

[36] Rorsman P, Huising MO. The somatostatin-secreting pancreatic δ-cell in health and disease. Nat Rev Endocrinol. 2018;14(7):404‐414. 29773871 10.1038/s41574-018-0020-6 PMC5997567

[37] Finan B, Capozzi ME, Campbell JE. Repositioning glucagon action in the physiology and pharmacology of diabetes. Diabetes. 2020;69(4):532‐541. 31178432 10.2337/dbi19-0004 PMC7085250

[38] Svendsen B, Larsen O, Gabe MBN, et al Insulin secretion Depends on intra-islet glucagon signaling. Cell Rep. 2018;25(5):1127‐1134.e2. 30380405 10.1016/j.celrep.2018.10.018

[39] Ito A, Horie I, Miwa M, et al Impact of glucagon response on early postprandial glucose excursions irrespective of residual β-cell function in type 1 diabetes: a cross-sectional study using a mixed meal tolerance test. J Diabetes Investig. 2021;12(8):1367‐1376. 10.1111/jdi.13486 PMC8354509 33369175

[40] Capozzi ME, Svendsen B, Encisco SE, et al β cell tone is defined by proglucagon peptides through cAMP signaling. JCI Insight. 2019;4(5):e126742. 30720465 10.1172/jci.insight.126742 PMC6483521

[41] Wojtusciszyn A, Armanet M, Morel P, Berney T, Bosco D. Insulin secretion from human beta cells is heterogeneous and dependent on cell-to-cell contacts. Diabetologia. 2008;51(10):1843‐1852. 18665347 10.1007/s00125-008-1103-z

[42] Hare KJ, Vilsbøll T, Holst JJ, Knop FK. Inappropriate glucagon response after oral compared with isoglycemic intravenous glucose administration in patients with type 1 diabetes. Am J Physiol Endo-crinol Metab. 2010;298(4):E832‐E837. 10.1152/ajpendo.00700.2009 20103744

[43] Greenbaum CJ, Prigeon RL, Alessio DAD. Impaired -cell function, incretin effect, and glucagon suppression in patients with type 1 diabetes who have normal fasting glucose. Diabetes. 2002;51(4):951‐957. 11916912 10.2337/diabetes.51.4.951

[44] Nauck M, Stockmann F, Ebert R, Creutzfeldt W. Reduced incretin effect in type 2 (non-insulin-dependent) diabetes. Diabetologia. 1986;29(1):46‐52. 3514343 10.1007/BF02427280

